# Measles Update — United States, January 1–April 17, 2025

**DOI:** 10.15585/mmwr.mm7414a1

**Published:** 2025-04-24

**Authors:** Adria D. Mathis, Kelley Raines, Thomas D. Filardo, Nicole Wiley, Jessica Leung, Paul A. Rota, Diana Martinez, Saroj Rai, Varun Shetty, Nora Holzinger, Emma Stanislawski, Demetre C. Daskalakis, Kevin Chatham-Stephens, Manisha Patel, David Sugerman

**Affiliations:** ^1^Division of Viral Diseases, National Center for Immunization and Respiratory Diseases, CDC; ^2^ASRT, Inc., Smyrna, Georgia; ^3^Texas Department of State Health Services; ^4^New Mexico Department of Health; ^5^Office of the Director, National Center for Immunization and Respiratory Diseases, CDC; ^6^Immunization Services Division, National Center for Immunization and Respiratory Diseases, CDC.

**Summary**

**What is already known about this topic?**

Although measles was declared eliminated in the United States in 2000, large outbreaks with 50 or more cases have become more frequent, especially in close-knit communities with low vaccination coverage.

**What is added by this report?**

During January 1–April 17, 2025, a total of 800 measles cases were reported in the United States, the second highest annual case count in 25 years; 82% were associated with an ongoing outbreak in close-knit communities with low vaccination coverage in New Mexico, Oklahoma, and Texas. Eighty-five (11%) patients were hospitalized, and three have died.

**What are the implications for public health practice?**

To prepare for and prevent measles cases and outbreaks, health departments should work with trusted messengers on culturally competent community engagement, education, vaccination efforts, and other infection prevention approaches. Increasing national and local measles, mumps, and rubella vaccination coverage is essential to preventing measles cases and outbreaks.

## Abstract

A multistate measles outbreak, predominantly affecting members of close-knit communities with low measles vaccination coverage in New Mexico, Oklahoma, and Texas began in January 2025. As of April 17, a total of 800 cases have been reported in the United States in 2025; 654 (82%) cases in New Mexico, Oklahoma, and Texas have been associated with the ongoing outbreak. These cases represent an approximately 180% increase over the 285 measles cases reported in the United States during all of 2024, and the second highest annual case count in the United States in 25 years. Overall, 771 (96%) patients have been unvaccinated or had unknown vaccination status (77% were unvaccinated, and 14% had unknown vaccination status when excluding 590 cases reported by Texas, which requires explicit consent by law [i.e., opt-in] to enroll in the Texas Immunization Registry), 85 (11%) patients have been hospitalized, and three patients have died. Among 48 (6%) internationally imported cases, 44 (92%) occurred among U.S. residents. Endemic measles was declared eliminated in the United States in 2000 as a direct result of high 2-dose childhood coverage with the measles, mumps, and rubella (MMR) vaccine. However, measles cases and outbreaks continue to occur when travelers with measles return to the United States while they are infectious; larger U.S. outbreaks typically follow importation into close-knit communities with low vaccination coverage. Nationally, risk for widespread measles transmission remains low because of high population-level immunity. To prepare for and prevent measles cases and outbreaks, public health departments should continue working with trusted community messengers on culturally competent community engagement, education, vaccination efforts, and other community infection prevention approaches (e.g., case isolation, contact monitoring, and post-exposure prophylaxis) and coordinating with health care facilities and schools. Increasing national and local MMR vaccination coverage is essential to preventing measles cases and outbreaks.

## Introduction

Measles is the most highly contagious febrile rash illness, infecting up to 90% of susceptible close contacts and resulting in serious complications such as pneumonia, encephalitis, and death. Among the 4,056 measles cases reported in the United States during 2001–2022, a total of 727 (18%) were hospitalized, and three deaths were reported*; of the 727 hospitalized patients, 473 (65%) were unvaccinated, and 187 (26%) had unknown vaccination status (*1*). Worldwide, measles vaccination is estimated to have saved 93.7 million lives during 1974–2024 and played a substantial role in reducing childhood mortality (*2*) by preventing complications associated with measles and deaths from other infectious diseases as a consequence of measles-related immunosuppression (*3*).

Endemic measles transmission was declared eliminated^†^ in the United States in 2000 after a change from a 1-dose to a 2-dose measles, mumps, and rubella (MMR) vaccination schedule in 1989 (*4*). However, a recent resurgence in global measles, resulting from COVID-19 pandemic–related challenges in implementing measles vaccination routine services and campaigns, has increased the risk for imported cases and outbreaks in the United States, particularly when U.S. travelers are exposed to measles abroad and return to the United States while they are infectious (*5*). Although the United States still benefits from high population immunity from routine MMR vaccination, declining immunization rates among school-aged children and communities with already low vaccination coverage threaten a resurgence of measles, along with its potentially serious associated complications. For this report, CDC used national surveillance data to describe the epidemiology of measles cases and outbreaks reported in the United States during the first 16 weeks of 2025.

## Methods

### Data Source and Case Classification

State health departments notify CDC of confirmed measles cases^§^ (*6*) through the National Notifiable Diseases Surveillance System and directly (by email or telephone) to the National Center for Immunization and Respiratory Diseases. Measles vaccination status is ascertained by health departments during each case investigation; patients with written or electronic documentation of receipt of ≥1 dose of a measles-containing vaccine ≥14 days before rash onset are considered vaccinated, and all other patients are classified as unvaccinated or as having unknown measles vaccination status.^¶^ Measles cases are classified by the Council of State and Territorial Epidemiologists as internationally imported if 1) at least part of the exposure period (7–21 days before rash onset) occurred outside the United States, 2) rash onset occurred within 21 days of entering the United States, and 3) no known exposure to measles occurred in the United States. All other cases are classified as U.S.-acquired (*6*). For this analysis, patients with imported measles cases were classified as age-eligible for vaccination if they were aged ≥6 months and were not vaccinated according to Advisory Committee on Immunization Practices (ACIP) recommendations (*4*).

### Analysis of Outbreaks

A measles outbreak was defined as the occurrence of three or more epidemiologically linked** cases. Unique measles virus sequences are defined as those differing by at least one nucleotide in the N-450 sequence (i.e., the 450 nucleotides encoding the carboxyl-terminal 150 nucleoprotein amino acids) based on standard World Health Organization recommendations for describing sequence variants^††^ (*7*). Patients with confirmed vaccine reactions (i.e., rash caused by a reaction to vaccine strain virus) were not included as persons with measles cases, as studies have found no confirmed instances of human-to-human transmission of the measles vaccine strain virus (*6*). This activity was reviewed by CDC, deemed not research, and was conducted consistent with applicable federal law and CDC policy.^§§^

## Results

### Characteristics of Reported Measles Cases

**FIGURE 1:**
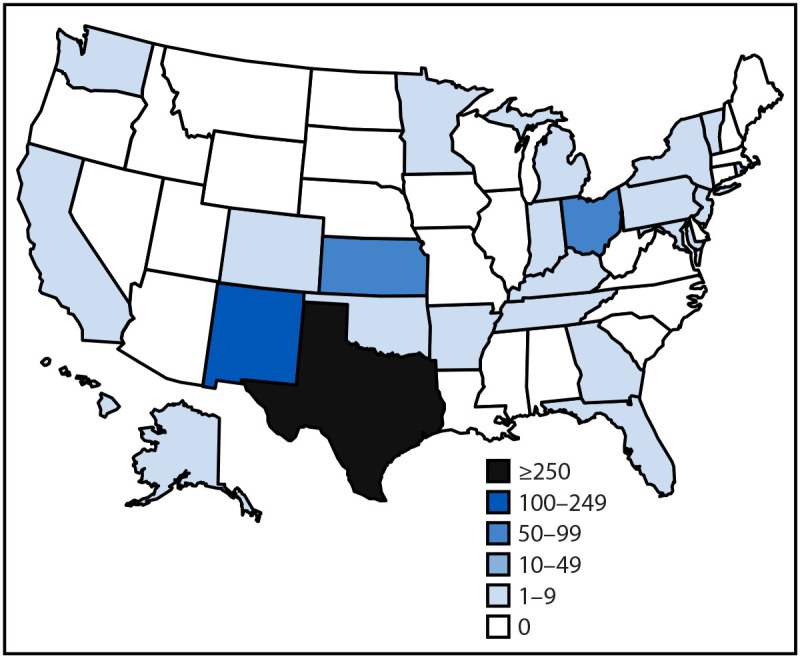
Reported number of confirmed* measles cases, by state (N = 800) — United States, January 1–April 17, 2025 * An acute febrile rash illness with laboratory confirmation of measles or a direct epidemiologic link to a laboratory-confirmed measles case.

**FIGURE 2:**
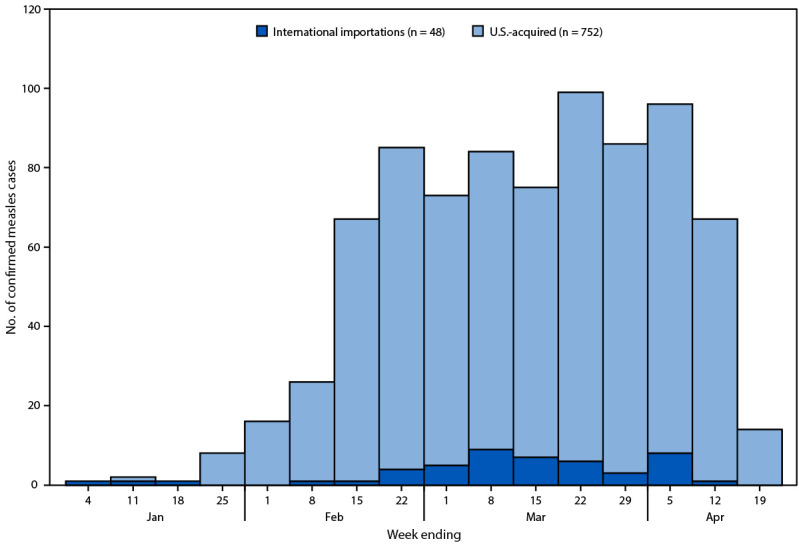
Number of reported confirmed* measles cases, by week of rash onset and importation status (N = 800) — United States, January 1–April 17, 2025^†^ * An acute febrile rash illness with laboratory confirmation of measles or a direct epidemiologic link to a laboratory-confirmed measles case. ^†^ Data are preliminary as of April 17, 2025. Data for the week ending April 19, 2025, are for a partial week.

During January 1–April 17, 2025, a total of 800 confirmed measles cases were reported in 25 U.S. jurisdictions (Figure 1). The highest number of weekly cases (99) was reported during the week ending March 22 (Figure 2). Median patient age was 9 years (IQR = 4–23 years); 249 (31%) patients were aged <5 years, 304 (38%) were aged 5–19 years, 231 (29%) were aged ≥20 years, and age was unknown for 16 (2%) patients (Table). Among all measles patients, 771 (96%) were unvaccinated or their vaccination status was unknown, 10 (1%) had received 1 dose of MMR vaccine, and 19 (2%) had received 2 doses. For Texas cases, it was not possible to disaggregate unvaccinated patients from those with unknown vaccination status because the Texas Immunization Registry requires explicit consent by law (i.e., opt-in) to enroll. Among 210 measles patients (excluding 590 cases reported by Texas), 162 (77%) were unvaccinated, six (3%) had received 1 dose of MMR vaccine, 12 (6%) had received 2 doses, and the vaccination status of 30 (14%) was unknown. Among all 800 cases, 790 (99%) occurred among U.S. residents. Overall, 85 (11%) patients were hospitalized; 56 (66%) of those were unvaccinated, one (1%) had received 1 dose of MMR vaccine, and the vaccination status of 28 (33%) was unknown. Three measles deaths were reported to CDC; two confirmed in Texas in unvaccinated school-aged children with no known underlying medical conditions, and one confirmed in New Mexico in an unvaccinated adult. Most cases (557; 70%) were laboratory-confirmed; among 251 (31%) cases from which specimens were available for molecular sequencing, all were confirmed as wild-type virus strain with 225 (90%) identified as genotype D8 and 26 (10%) as genotype B3. 

**TABLE:** Selected characteristics of patients with reported measles — United States, January 1–April 17, 2025*

Characteristic	No. of measles cases (%)		
	Total	International importations	U.S.-acquired
**Total measles cases**	**800 (100)**	**48 (6)**	**752 (94)**
**Age group, yrs**			
<5	**249 (31)**	17 (35)	232 (31)
5–19	**304 (38)**	6 (13)	298 (40)
≥20	**231 (29)**	22 (46)	209 (28)
Unknown	**16 (2)**	3 (6)	13 (2)
**Measles vaccination status**			
Unvaccinated or unknown	**771 (96)**	43 (90)	728 (97)
Vaccinated, 2 doses	**19 (2)**	4 (8)	15 (2)
Vaccinated, 1 dose	**10 (1)**	1 (2)	9 (1)
**Measles vaccination status (excluding Texas residents)**			
Unvaccinated	**162 (77)**	30 (68)	132 (80)
Unknown	**30 (14)**	9 (20)	21 (13)
Vaccinated, 2 doses	**12 (6)**	4 (9)	8 (5)
Vaccinated, 1 dose	**6 (3)**	1 (2)	5 (3)
**Residency**			
U.S. resident	**790 (99)**	44 (92)	746 (99)
**Outcome**			
Hospitalized	**85 (11)**	15 (31)	70 (9)
Died^†^	**3 (3.8)**	0 (—)	3 (4.0)
**Vaccination status of hospitalized patients** ^§^			
Unvaccinated	**56 (66)**	11 (73)	45 (64)
Unknown	**28 (33)**	3 (20)	25 (36)
Vaccinated, 1 dose	**1 (1)**	1 (7)	0 (—)

* Data are preliminary as of April 17, 2025.

^†^ Deaths per 1,000 persons with measles.

^§^ Percentage among all hospitalized patients.

### International Importations

Forty-eight (6%) cases were directly imported from other countries, including 44 (92%) among U.S. residents who had traveled abroad; 752 (94%) cases were U.S.-acquired. Fifteen (31%) importations resulted in secondary cases. Among the 48 internationally imported measles cases, 33 (69%) patients were unvaccinated, one (2%) had received 1 dose of MMR vaccine, four (8%) had received 2 doses, and the vaccination status of 10 (21%) patients was unknown. All 33 of the unvaccinated persons with imported measles were age-eligible for vaccination per ACIP, including 10 infant travelers aged 6–11 months. Source countries of the 48 imported measles cases included Canada (10 cases), Vietnam (10), Mexico (seven), Pakistan (three), the Philippines (two), Saudi Arabia (two), and one imported case each from Afghanistan, Australia, Guinea, Netherlands, Somalia, Spain, and Uganda; a source country could not be determined for seven travelers who visited multiple countries during their exposure period: Tanzania and United Arab Emirates (two cases); China, Japan, and Vietnam (one); France, South Korea, and Vietnam (one); Thailand and Vietnam (one); Indonesia and the Philippines (one); and Southeast Asia (one).

### Measles Outbreaks

Ten measles outbreaks have been reported in 2025^¶¶^; 751 (94%) of all reported confirmed measles cases were outbreak-associated. An imported source was identified for seven outbreaks, and the source of three outbreaks remains unknown. Outbreak-related cases have been reported in 12 states (Georgia, Indiana, Kansas, Kentucky, Michigan, New Jersey, New Mexico, Ohio, Oklahoma, Pennsylvania, Tennessee, and Texas). The largest outbreak began among a close-knit community with low vaccination coverage in Gaines County, Texas in January 2025 and has accounted for 654 (82%) cases reported during 2025 (584 patients in 24 Texas counties, 63 patients in four New Mexico counties, and seven patients in northeastern Oklahoma); the source of this outbreak remains unknown. Thirty-seven confirmed cases in Kansas are suspected to be linked to this outbreak. In addition, an expanding outbreak in Chihuahua, Mexico*** began in late February after a Mexican resident became infected after reported travel to Gaines County, Texas. All 208 genotyped specimens obtained from measles patients in Kansas, New Mexico, and Texas were genotype D8, 196 (94%) of which had identical N-450 sequences; 12 differed by one nucleotide, which can be expected in prolonged outbreaks.

## Discussion

A total of 800 measles cases and 10 outbreaks were reported in the United States during the first 16 weeks of 2025, representing approximately a 180% increase over the 285 measles cases reported in the United States during all of 2024. Most cases have been associated with an ongoing outbreak in close-knit communities with low vaccination coverage in New Mexico, Oklahoma, and Texas. 

Overall, 11% of measles patients have been hospitalized, and three deaths have been reported. Similar to previous years (*1*), nearly all (96%) cases occurred in persons who were unvaccinated or whose vaccination status was unknown, and 77% of cases occurred in persons who were unvaccinated when excluding cases reported by Texas. Most (92%) imported cases occurred among U.S. residents returning to the United States while infectious and from all six World Health Organization regions. Adherence to standard measles control measures, including isolation and quarantine, as well as high vaccination coverage locally, prevented secondary transmission from most of these persons who were infectious after returning from travel abroad.

Most cases reported during 2025 have been associated with an ongoing outbreak in close-knit communities in New Mexico, Oklahoma, and Texas, resulting in the second largest outbreak in the United States since elimination was declared in 2000. During 2001–2023, approximately 90% of U.S. measles outbreaks with 50 or more cases occurred in close-knit communities with low vaccination coverage (*8*). Such communities might have frequent communal gatherings and have concerns about engaging with public health and health care systems for testing, treatment, and vaccination. The United States, Canada,^†††^ and Mexico are all experiencing large, expanding outbreaks in similar interconnected communities. Frequent travel among similar communities across multiple states and countries might facilitate the rapid spread of measles outbreaks. The risk for widespread measles transmission in the United States remains low because of high population immunity resulting from high measles vaccination coverage. However, recent increasing global measles incidence in areas frequently visited by U.S. travelers, coupled with declines in MMR vaccination coverage in many U.S. jurisdictions to <95% (the estimated population-level immunity necessary to prevent measles outbreaks), and spread of measles from ongoing domestic outbreaks to other jurisdictions, have increased the risk for ongoing measles transmission within the United States (*8*,*9*).

### Limitations

The findings in this report are subject to at least four limitations. First, imported cases were likely underreported because 30% of reported outbreaks had no known source. Second, outbreak-related cases were likely underreported because certain persons in affected communities might not engage with the health care and public health systems. Third, distinguishing unvaccinated patients from patients with unknown measles vaccination status in Texas was not possible; the Texas Immunization Registry legally requires explicit consent, or opt-in, for adults and by parent or guardian for children to enroll.^§§§^ Persons with no records available are considered to have an unverified vaccination history. Finally, definitive linkages between the large outbreak in New Mexico, Oklahoma, and Texas and cases reported in Kansas could not be identified.

### Implications for Public Health Practice

To protect against measles and its complications before traveling internationally, all persons aged ≥12 months should have documented receipt of 2 appropriately spaced doses of MMR vaccine, and infants aged 6–11 months of age should receive 1 dose of MMR vaccine (*10*). Persons residing in or traveling domestically to outbreak areas should follow local public health guidance, which is developed based on review and analysis of the local outbreak epidemiology (*6*). Infants aged <6 months are at high risk for measles complications but are too young to be vaccinated, and therefore depend upon population immunity and passively transferred maternal measles antibodies (from previously vaccinated or infected mothers) to prevent infections and related complications.

Health care providers continue to serve on the front lines to identify measles cases, alert public health departments^¶¶¶^, ensure recommended testing, and implement measles isolation precautions to prevent health care–associated and community-based transmission. Health care providers should consider measles in the differential diagnosis for all patients (especially those who are unvaccinated) who 1) have fever (temperature ≥101°F [≥38.3°C]) and a generalized maculopapular rash with cough, coryza, or conjunctivitis, 2) have recently traveled outside the country or to a U.S. region with a known measles outbreak, or 3) have other known or suspected exposure to measles (*6*). Although no specific Food and Drug Administration–approved antiviral therapy for measles exists, rapid access to supportive care can help relieve symptoms and treat complications such as pneumonia and secondary bacterial and viral infections. Providers should also offer and encourage vaccination for eligible patients who lack presumptive evidence of immunity to measles (*4*).

Public health departments might benefit from using a CDC checklist**** to help guide their readiness activities such as preparing for laboratory testing and data reporting needs, conducting tabletop exercises, and facilitating early engagement with communities with low vaccination coverage and their trusted messengers before measles and other vaccine-preventable disease outbreaks occur. To identify communities at risk, public health departments should consider using both MMR vaccination coverage data from immunization information systems and kindergarten entry and vaccination exemption data from kindergarten entry records. Standard measles control interventions, including vaccination, isolation, quarantine, and postexposure prophylaxis (i.e., administration of MMR vaccine within 72 hours of exposure or immunoglobulin within 6 days of exposure for certain persons) (*10*), might be challenging to implement in certain communities. Therefore, public health departments should consider partnering with trusted community messengers (e.g., clinicians and religious leaders) on culturally competent community engagement, education, vaccination efforts, and potentially acceptable community infection control approaches. Coordination with health care facilities, early childhood education facilities and schools, and other congregate settings that surround or serve these communities to prepare for measles cases regarding appropriate infection prevention and control, testing, public health follow-up, and early childhood education or school exclusion policies is crucial to limit transmission. Increasing national and local MMR vaccination coverage is essential to preventing measles cases and outbreaks.

## Footnotes

* Two measles deaths were reported in 2003 (one in a child aged 13 years who had chronic granulomatous disease and one in an adult aged 75 years), and one was reported in 2015 in an adult with immunocompromise aged 28 years.

† Measles elimination is defined as the absence of endemic measles transmission for ≥12 months in the presence of an adequate surveillance system.

§ An acute febrile rash illness with laboratory confirmation (detection of measles virus–specific nucleic acid from a clinical specimen using real-time reverse transcription–polymerase chain reaction or a positive serologic test for measles immunoglobulin M antibody) or direct epidemiologic linkage to a laboratory-confirmed case.

¶ For residents of Texas, vaccination history is verified in the Texas Immunization Registry (ImmTrac2) or by review of vaccination records; patients with no vaccination records in the registry were considered to have an unverified vaccination history. Texas only disaggregates unvaccinated and unknown vaccination status among hospitalized patients; these records are provider-verified.

** Epidemiologic linkages include having known or suspected contact with an infectious measles patient during the exposure period (7–21 days before rash onset) and living in or visiting a geographic area with ongoing measles transmission during the exposure period.

†† Genotyping was performed at CDC and at the Vaccine Preventable Disease Reference Centers of the Association of Public Health Laboratories.

§§ 45 C.F.R. part 46.102(l)(2), 21 C.F.R. part 56; 42 U.S.C. Sect. 241(d); 5 U.S.C. Sect. 552a; 44 U.S.C. Sect. 3501 et seq.

¶¶ At the time of this report, two measles outbreaks have ended, and eight outbreaks are ongoing. A measles outbreak is considered to be over when no new cases have been identified during two incubation periods (42 days) since the rash onset in the last outbreak-related case.

*** https://www.gob.mx/cms/uploads/attachment/file/990598/Aviso_Epidemiologico_Sarampio_n__16_abril_2025.pdf

†††
https://health-infobase.canada.ca/measles-rubella/

§§§
https://www.cdc.gov/iis/policy-legislation/texas.html

¶¶¶
https://libraries.cste.org/after-hours-contact/

****
https://www.cdc.gov/measles/media/pdfs/2025/02/CDC-Public-Health-Checklist_Sept18_FINAL-updatedlinks-508.pdf

## References

[1] Leung J, Munir NA, Mathis AD, The effects of vaccination status and age on clinical characteristics and severity of measles cases in the United States in the postelimination era, 2001–2022. Clin Infect Dis 2025;80:663–72. 10.1093/cid/ciae470 39271123 PMC11955208

[2] Shattock AJ, Johnson HC, Sim SY, Contribution of vaccination to improved survival and health: modelling 50 years of the Expanded Programme on Immunization. Lancet 2024;403:2307–16. 10.1016/S0140-6736(24)00850-X 38705159 PMC11140691

[3] Mina MJ, Metcalf CJ, de Swart RL, Osterhaus AD, Grenfell BT. Long-term measles-induced immunomodulation increases overall childhood infectious disease mortality. Science 2015;348:694–9. 10.1126/science.aaa3662 25954009 PMC4823017

[4] McLean HQ, Fiebelkorn AP, Temte JL, Wallace GS; CDC. Prevention of measles, rubella, congenital rubella syndrome, and mumps, 2013: summary recommendations of the Advisory Committee on Immunization Practices (ACIP). MMWR Recomm Rep 2013;62(No. RR-4):1–34. 23760231

[5] Minta AA, Ferrari M, Antoni S, Progress toward measles elimination—worldwide, 2000–2023. MMWR Morb Mortal Wkly Rep 2024;73:1036–42. 10.15585/mmwr.mm7345a4 39541251 PMC11576049

[6] Filardo TD, Mathis A, Raines K, Measles [Chapter 7]. In: Manual for the surveillance of vaccine-preventable diseases. Atlanta, GA: US Department of Health and Human Services, CDC; 2024. https://www.cdc.gov/surv-manual/php/table-of-contents/chapter-7-measles.html

[7] Williams D, Penedos A, Bankamp B, Update: circulation of active genotypes of measles virus and recommendations for use of sequence analysis to monitor viral transmission. Wkly Epidemiol Rec 2022;97:485–92. https://reliefweb.int/report/world/weekly-epidemiological-record-wer-30-september-2022-vol-97-no-39-2022-pp-481-492-enfr

[8] CDC. Assessing measles outbreak risk in the United States. Atlanta, GA: US Department of Health and Human Services; 2024. https://www.cdc.gov/ncird/whats-new/measles-outbreak-risk-in-us.html

[9] Seither R, Yusuf OB, Dramann D, Coverage with selected vaccines and exemption rates among children in kindergarten—United States, 2023–24 school year. MMWR Morb Mortal Wkly Rep 2024;73:925–32. 10.15585/mmwr.mm7341a3 39418212 PMC11486350

[10] CDC. Vaccines and immunizations: routine measles, mumps, and rubella vaccination. Atlanta, GA: US Department of Health and Human Services, CDC; 2021. https://www.cdc.gov/vaccines/vpd/mmr/hcp/recommendations.html

